# Randomized controlled trial of a theory-informed mHealth intervention to support ART adherence and viral suppression among women with HIV in Mombasa, Kenya: preliminary efficacy and participant-level feasibility and acceptability

**DOI:** 10.1186/s12889-023-15638-3

**Published:** 2023-05-08

**Authors:** Frances M. Aunon, George Wanje, Barbra A. Richardson, Linnet Masese, Thomas A. Odeny, John Kinuthia, Kishorchandra Mandaliya, Walter Jaoko, Jane M. Simoni, R. Scott McClelland

**Affiliations:** 1 Department of Psychology, University of Washington, Seattle, WA United States of America; 2 Department of Medical Microbiology, University of Nairobi, Nairobi, Kenya; 3 Department of Biostatistics, University of Washington, Seattle, WA United States of America; 4 Department of Global Health, University of Washington, Seattle, WA United States of America; 5 Department of Medicine, University of Washington, Seattle, WA United States of America; 6 Department of Medicine, University Washington in St. Louis, St. Louis, MO United States of America; 7 Department of Research & Programs, Kenyatta National Hospital, Nairobi, Kenya; 8 Department of Gender, Women and Sexuality Studies, University of Washington, Seattle, WA United States of America; 9 Department of Epidemiology, University of Washington, Seattle, WA United States of America; 10 VA Connecticut Healthcare System, West Haven, CT United States of America; 11 Department of Psychiatry, Yale School of Medicine, New Haven, CT United States of America

**Keywords:** ART, Viral suppression, Adherence, Intervention, Sex workers, Sub-saharan Africa, Randomized controlled trial

## Abstract

**Background:**

Mobile Health (“mHealth”) interventions have shown promise in improving HIV treatment outcomes for stigmatized populations. This paper presents the findings from a randomized controlled trial to assess the efficacy, participant-level feasibility and acceptability of a theory-informed mHealth intervention, *Motivation Matters!*, designed to improve viral suppression and ART adherence among HIV-seropositive women who engage in sex work in Mombasa, Kenya.

**Methods:**

A total of 119 women were randomized between the intervention and standard of care control. The primary outcome examined viral suppression (≤ 30 copies/mL) six months following ART initiation. ART adherence was assessed monthly using a visual analogue scale. Participant-level feasibility was measured through response rates to study text messages. Acceptability was assessed through qualitative exit interviews.

**Results:**

Six months following treatment initiation, 69% of intervention and 63% of control participants were virally suppressed (Risk Ratio [RR] = 1.09, 95% Confidence Interval [95% CI] (0.83, 1.44). Among women who were viremic at baseline and endorsed engagement in sex work, 74% of women in the intervention arm compared with 46% of women in the control arm achieved viral suppression at month six RR = 1.61, 95% CI (1.02, 2.55). Adherence was higher in intervention versus control participants every month. All participants responded to at least one message, and there was a 55% overall response rate to intervention text messages. Qualitative exit interviews suggested high acceptability and perceived impact of the intervention.

**Conclusion:**

The improvements in ART adherence and viral suppression, combined with encouraging data on feasibility and acceptability, provides preliminary evidence that *Motivation Matters!* could support ART adherence and viral suppression in women who engage in sex work.

**Trial registration:**

This trial was registered with ClinicalTrials.gov (NCT02627365, 10/12/2015; http://clinicaltrials.gov).

## Background

In 2016, the Joint United Nations Programme on HIV/AIDS established three interrelated benchmarks for HIV care; by 2030, 95% of people with HIV (PWH) would know their HIV status, 95% of people who know their HIV status would be on antiretroviral therapy (ART), and 95% of people on ART would have suppressed viral loads [1]. Identifying strategies to improve viral suppression, especially in regions and populations most affected by HIV, could have a substantial impact on the epidemic.

Sub-Saharan Africa is home to 10% of the world’s population but contains nearly 70% of the global population of PWH [1]. Women in sub-Saharan Africa bear a disproportionate burden of HIV due to intersecting economic, structural, and biological factors [2]. African females who engage in sex work (FSW) are at elevated risk for acquiring and transmitting HIV [3], highlighting the need for innovative interventions to support HIV treatment and prevention in this population.

The ubiquity of mobile technology in sub-Saharan Africa provides unique opportunities for mHealth interventions. These interventions, which include short message service (SMS) and tablet-based applications (“apps”), have been leveraged to support ART adherence and viral suppression [4], and have generally been highly acceptable to providers and patients [5, 6]. Moreover, a recent review highlighted that mHealth interventions can be more rigorous and impactful when derived using a theoretical framework [7]. Theory-driven mHealth interventions may be particularly useful in targeting stigmatized populations [5, 8–10], but few have been developed by and specifically for FSW [11].

The objective of this randomized controlled trial (RCT) was to test a culturally-tailored, Information-Motivation-Behavioral Skills theory-informed (IMB) [12], mHealth intervention designed to improve viral suppression among FSW. This intervention, entitled *Motivation Matters!*, was designed to support adherence in FSW initiating ART or changing regimens due to virologic failure. It was hypothesized that the individualized, 2-way mHealth intervention would significantly increase HIV viral suppression compared to control. Additionally, we evaluated the intervention’s feasibility by examining participant engagement with study text messages and acceptability through qualitative exit interviews.

## Methods

This study evaluated the efficacy of an mHealth intervention versus standard of care control for improving viral suppression (≤ 30 copies/mL) six months after ART initiation or regimen change in a two-arm randomized controlled trial. Participants were allocated in a 1:1 ratio. The goal was to enroll 210 women. In the course of enrollment, it became clear that the population was much harder than anticipated to recruit at a single site. In light of this limitation, the focus of the study was shifted to providing a preliminary measure of efficacy in combination with an evaluation of participant-level feasibility and acceptability to inform a future multi-site trial. The methods presented here reflect the study as implemented.

### Participants

Women were recruited from the Mombasa Cohort, a long-term open cohort study of FSW in Mombasa, Kenya [13]. Women were eligible to participate if they were ≥ 18 years old or an emancipated minor, HIV-seropositive, initiating ART or starting a new regimen following treatment failure, self-identified as exchanging sex for cash or in-kind payment, had access to a mobile phone, were willing to receive text messages, and were able to read or had a trusted confidant who could read messages to them. Women were excluded if they had plans to move away in the next six months or had a contraindication to immediate ART initiation.

After nine months, due to slow enrollment, the inclusion criteria were expanded to include women who did not engage in sex work. This change was possible because the messages focused on information, motivation, and behavioral skills for ART adherence, and were not specific to the context of transactional sex. General population women were recruited from voluntary counselling and testing centers and met all other eligibility criteria.

### Ethical approvals and consent for participation

All research procedures were approved by the Kenyatta National Hospital - University of Nairobi Ethics (P102/02/2015) and Research Committee and the Human Subjects Research Committee of the University of Washington. All participants provided written informed consent.

### Procedures

Participants were recruited from the Mombasa Cohort, a long-term open cohort study of women who self-identified as having exchanged sex for goods or money in the past month at the time of enrollment. Participants in the Mombasa Cohort attended monthly follow-up appointments with regular HIV and STI screening. Detailed methods have been described elsewhere [14]. Mombasa Cohort participants who were HIV-positive but had not started ART were eligible to screen for the *Motivation Matters!* trial. No repeat HIV testing was required for women who were switching ART regimens due to treatment failure.

Eligible women were offered enrollment after completing written informed consent for participation in the trial. A face-to-face interview was conducted using a standardized questionnaire to collect information on demographic characteristics, medical history, and sexual behavior. Participants received a second pre-ART counseling session that highlighted the importance ART adherence and discussed barriers and facilitators. A one-month supply of ART was dispensed, and participants were randomized to the intervention and control conditions.

The randomization was generated by a biostatistician who had no other role in the study. Participants were assigned to the intervention or control arm in a 1:1 ratio using block randomization with variable block sizes. In Mombasa, treatment groups were assigned using sealed, opaque, serially numbered envelopes ordered in the sequence of treatment assignments generated using STATA version 14 (StataCorp, College Station, TX, USA). Due to the nature of the intervention, neither participants nor study staff could be blinded to study arm assignment. Differences in clinical management and counselling were minimized by providing pre-ART counseling prior to randomization and adhering to comprehensive standard operating procedures that guided participant counseling and evaluations during follow-up.

With the exception of informed consent forms, hard copies of data collection forms and electronic files did not include personal identifiers. All study documents were maintained in locked cabinets within filing rooms accessible only to study staff, and consents were stored separately from other study documents. Electronic data files were encrypted, password protected and accessible only to the research team.

Following the enrollment visit, all participants were asked to return monthly for six months. At each visit, study staff assessed ART adherence, discussed adherence barriers, and provided ART refills. Adherence data were collected monthly using a self-reported, validated visual analogue scale (VAS) [15, 16]. Adverse events were assessed at each visit. At months three and six, participants had blood drawn for CD4 count and plasma viral load. Viral loads for all samples were batched and tested after completion of follow-up. At month six, participants completed a semi-structured interview to collect qualitative feedback about their experiences. This interview included questions about women’s motivation for adherence and interactions with healthcare providers. The interviewer also administered the LifeWindows Information–Motivation–Behavioral Skills ART Adherence Questionnaire (LW-IMB-AAQ), a 33-item measure designed to assess information, motivation, and behavioral constructs related to ART adherence [17–20]. Participants received 250 Kenya Shillings (approximately US$2.50) at each visit as compensation for transportation costs.

Participants randomized to the control arm received standard of care for ART delivery according to Kenyan guidelines. Consistent with the standard of care for ART delivery in the research clinic, participants in the control arm were provided with a phone number to contact a nurse or doctor if they had questions.

In addition to all services provided in the control condition, participants randomized to the intervention arm received the mHealth intervention, *Motivation Matters!*. The intervention was informed by iterative focus group discussions (FGDs) with FSW with HIV. In accordance with IMB theory [12], the intervention included text messages related to information, motivation, and behavioral skills to support ART adherence. The intervention development has been published and includes the full text of all intervention messages [21]. Women received three messages per week in month one, then two messages per week until study completion. To maintain privacy, HIV was referred to “blood pressure” and ART as “medicine.” Earlier messages included more informational (e.g. “Hi [name], For blood pressure to be controlled, take all of your medicine at the same time every day. How are you doing? Sister Carol”) and motivational content (e.g. “[Hi [name], Your children are precious. Continue taking good care of yourself, so that you can take care of them. Are you well? Sister Carol”), while later messages included more behavioral skills (e.g. “Hi [name], A reminder such as alarms, start of TV shows, or before bed can help you take your blood pressure medicine. Any questions? Sister Carol”). Messages were personalized based on participants’ name, preferred language (English or Kiswahili), religion (Christian, Muslim, or neither), parity, and delivery time. Messages were automatically sent via the TextIt platform [22, 23]. Women were instructed to respond to text messages with either “poa” (“okay”) or “swali” (“question”), utilizing the same response options as an earlier trial of an mHealth intervention to support ART adherence in Kenya [8]. A study nurse called women who indicated they had a question within one business day. If participants did not reply to text messages within 48 h, they received a reminder message. If there was still no response, study staff made a follow-up call. To cover the 1 Kenyan shilling (approximately US$0.01) cost per message of responding to 8–12 study text messages each month, women in the intervention arm received pre-paid airtime credit of 50 Kenyan shillings (approximately US$5) at each visit.

During the exit interview six months following treatment initiation, we assessed the intervention’s acceptability. Women in the intervention arm were asked to share their reactions to the intervention and suggestions for improvement. Specifically, feedback was elicited about the intervention’s content, structure (including the number and frequency of messages), and confidentiality concerns.

Results were disseminated to participants and stakeholders through a Community Advisory Board and multiple dissemination meetings targeting different groups including participants, collaborating institutions, and health department officials at the local and national level.

### Laboratory

All assays were performed in the research laboratory in Mombasa. Screening and confirmatory HIV tests were performed according to Kenyan guidelines [24], using Determine (Unipath Limited, Bedford, UK) and Unigold (Trinity Biotech, Jamestown NY, USA) kits. Enumeration of CD4 cells was performed by FACSCount (BD Biosciences, Erembodegem, Belgium). Plasma HIV viral loads were quantified using the Aptima system (Hologic Corporation, San Diego, CA, USA).

### Sample size and statistical analysis

Based on prior research in this population [25], it was estimated that 75% of women in the control arm would have an undetectable viral load at month six. Assuming 90% viral suppression in the intervention arm, a sample size of 100 women per arm was needed to reject the null hypothesis that viral suppression did not differ significantly in the intervention and control arms with 80% power for a two-sided, uncorrected chi-squared statistic, at a significance level of 0.05. The sample size estimate anticipated that 95% of participants would contribute to the primary analysis. Women who were lost to follow-up were considered to be not virally suppressed and included in the analyses. Thus, the trial aimed to recruit 210 women (105 per arm). This trial was registered with ClinicalTrials.gov (NCT02627365; 10/12/2015; http://clinicaltrials.gov).

All data were collected on hard copy case report forms and entered in an electronic database with internal logic checks for key variables. Line listing of all variables was performed by printing out a hard copy of the electronic dataset and manually comparing it to the source documents to identify and correct any key-in errors.

The primary analysis comparing viral suppression (≤ 30 copies/mL) at month six was performed according to the intent-to-treat principle. A chi-squared test was used to compare the proportion of participants with suppressed viral load vs. detectable viral load in the intervention arm compared to the control arm. Participants who actively withdrew or were known to have died were excluded from analyses. Participants who were lost to follow-up contributed to final endpoint analyses by assuming that they had a detectable viral load at six months. Generalized linear models with a log-binomial link were used to examine the effect of the intervention on viral suppression and having 100% or <100% self-reported adherence each month [26]. T-tests were performed to examine differences in the information, motivation, and behavior subscales of the LW-IMB-AAQ between women randomized to the intervention versus control arms.

Participant-level feasibility was measured by the proportion of participants who responded to text messages, the overall proportion of messages receiving a response, and the proportion of clients who asked a question.

Participant-level intervention acceptability was assessed through content analysis of qualitative exit interviews. Interviews were audio-recorded, transcribed, and coded by authors GW and FMA. Content analysis using an inductive approach was used to identify emergent themes.

## Results

**Fig. 1:**
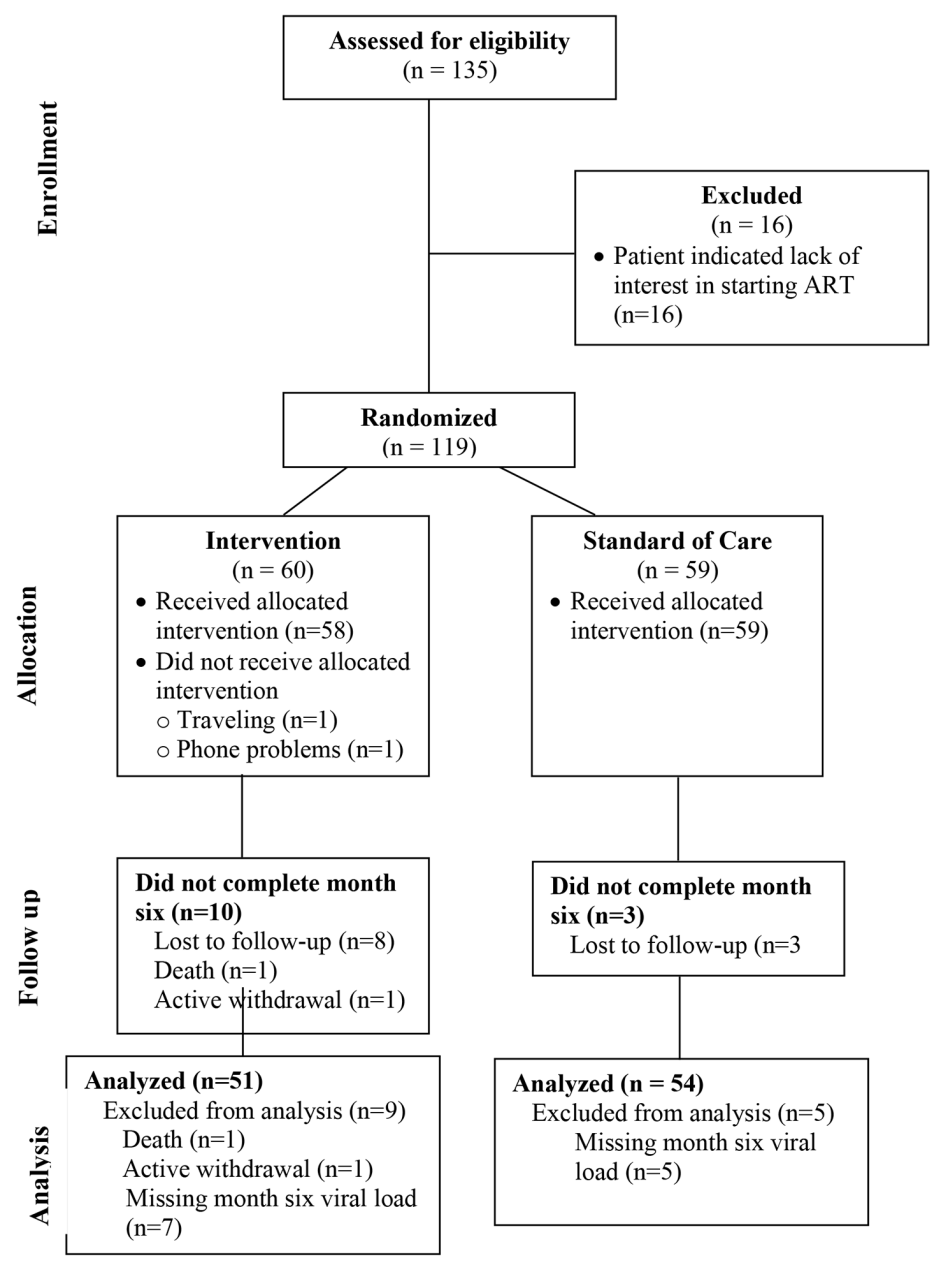
Consort Diagram for *Motivation Matters!* Randomized Controlled Trial

Between July 2016 and December 2017, 135 women were screened, of whom 119 (88%) were enrolled (Fig. 1). All 16 women who were not enrolled declined ART initiation (16/135, 12%). Sixty participants were randomized to the intervention condition and 59 were randomized to the standard of care control. One participant died during follow-up, cause unknown, and one participant voluntarily withdrew because she was moving out of the region. Twelve women were lost to follow-up. HIV RNA viral load test results for month six were compromised for 12 participants who were retained through the six-month intervention period; their data was excluded from the primary outcome analysis.

Participants had a mean age of 33.9 (standard deviation [*SD*] = 8.1) years and a mean of 8.9 years of education (*SD* = 3.4; Table 1). Of the 119 women enrolled, 108 (91%) reported that they were newly initiating ART and 11 (9%) were switching regimens due to treatment failure. Baseline plasma viral load was undetectable in 23 (20%) women. Current engagement in sex work was reported by 88 (74%) participants. At enrollment, 97 (82%) women reported sexual activity during the past week, of whom 56 (62%) reported consistent condom use.

**Table 1:** Enrollment socio-demographic and clinical characteristics by study condition

Variable	All (*N* = 119) Mean (*SD*) or % (*n*)	Intervention (*n =* 60) Mean (*SD*) or % (*n*)	Control (*n =* 59) Mean (*SD*) or % (*n*)
Age (Mean, SD)	33.9 (8.1)	33.6 (8.0)	34.2 (8.2)
Marital Status: % (n)			
Married Never Married Widowed/Divorced	10.9 (13) 21.0 (25) 58.0 (69)	5.0 (3) 21.7 (13) 61.7 (37)	16.9 (10) 20.3 (12) 54.2 (32)
Years of School Mean (SD)	8.9 (3.4)	8.6 (3.2)	9.2 (3.6)
Religion			
Christian % (n) Muslim % (n) Other % (n)	69.7 (83) 14.3 (17) 0.8 (1)	68.3 (41) 13.3 (8) 1.7 (1)	71.2 (42) 15.3 (9) 0.0 (0)
Any Alcohol Use % (n)	68.1 (81)	73.3 (44)	62.7 (37)
In past week:			
Condomless sex Abstinent	29.4 (35) 18.5 (22)	31.7 (19) 13.3 (8)	27.1 (16) 23.7 (14)
If had sex in past week:			
100% condom # sex acts (Median, IQR) # sex partners (Median, IQR)	61.5 (56) 2.0 (1.0, 5.0) 2.0 (1.0, 5.0)	62.0 (31) 3.0 (1.0, 5.3) 2.0 (1.0, 5.3)	61.0 (25) 2.0 (1.0, 3.0) 1.0 (1.0, 3.0)
Transactional sex	73.9 (88)	85.0 (51)	62.7 (37)
Viral load (copies/mL)	96464 (210804)	93832 (221264)	99095 (201703)
Viral load ≤ 30 copies/mL	19.8 (23)	19.0 (11)	20.7 (12)

At the primary endpoint analysis at six months, viral suppression was observed in 35/51 (69%) women in the intervention arm compared to 34/54 (63%) women in the control arm (Table 2; Risk Ratio [RR] 1.09 95% Confidence Interval [95% CI] 0.83 1.44). Secondary analyses were performed excluding the 19/105 (18%) participants with suppressed baseline viral load and 14/105 (13%) women who were not engaged in sex work. Of the remaining women, 26/35 (74%) in the intervention arm and 12/26 (46%) in the control arm achieved viral suppression at month six, (RR 1.61, 95% CI 1.02, 2.55).

**Table 2:** Viral suppression (≤ 30 copies/mL) following six month intervention period

	Intervention % (*n/N*)	Control % (*n/N*)	Relative Risk (RR)	95% CI for RR	*p-*value*
All women	68.6 (35/51)	63.0 (34/54)	1.09	0.83, 1.44	0.52
FSW	78.6 (33/42)	63.2 (24/38)	1.24	0.93, 1.66	0.10
Viremic at Baseline	73.7 (28/38)	59.5 (22/37)	1.24	0.89, 1.72	0.50
FSW viremic at Baseline	74.3 (26/35)	46.2 (12/26)	1.61	1.02, 2.55	0.04

*Relative Risk from log binomial generalized linear model

Perfect adherence by VAS was higher in intervention participants compared to control participants during every month of the intervention. The greatest difference, and the only one that was statistically significant, was adherence in the first month following initiation of the ART regimen (53/57 [93%] versus 37/52 [71%], RR 1.31, 95% CI 1.08, 1.58; Table 3).

**Table 3:** Adherence outcomes over the six month intervention period by study condition

	All % (*n/N*)	Intervention % (*n/N*)	Control % (*n/N*)	Relative Risk (RR)	95% CI for RR	*p-*value*
Perfect Adherence by VAS						
Month 1 Month 2 Month 3 Month 4 Month 5 Month 6	82.6 (90/109) 88.0 (88/100) 89.8 (88/98) 86.0 (86/100) 83.5 (81/97) 83.5 (81/97)	93.0 (53/57) 88.2 (45/51) 90.2 (46/51) 88.0 (44/50) 89.8 (44/49) 87.2 (41/47)	71.2 (37/52) 87.8 (43/49) 89.4 (42/47) 84.0 (42/50) 77.1 (37/48) 80.0 (40/50)	1.31 1.01 1.01 1.05 1.16 1.09	1.08, 1.58 0.87, 1.16 0.88, 1.15 0.89, 1.23 0.97, 1.40 0.91, 1.30	0.01 0.91 0.90 0.59 0.09 0.32

*Relative Risk from log binomial generalized linear model.

Relative to women in the control arm, women in the intervention arm endorsed higher values on the LW-IMB-AAQ [16], though none of the differences were statistically significant. Specifically, women reported having more information about HIV (88.3 [*SD =* 15.5] versus 85.3 [*SD =* 19.6]), motivation to adhere to ART (70.8 [*SD = 17.6*] versus 67.3 [*SD =* 23.1]), and behavioral skills for ART adherence (80.8 [*SD =* 15.4] versus 77.0 [*SD =* 18.7]).

**Fig. 2:**
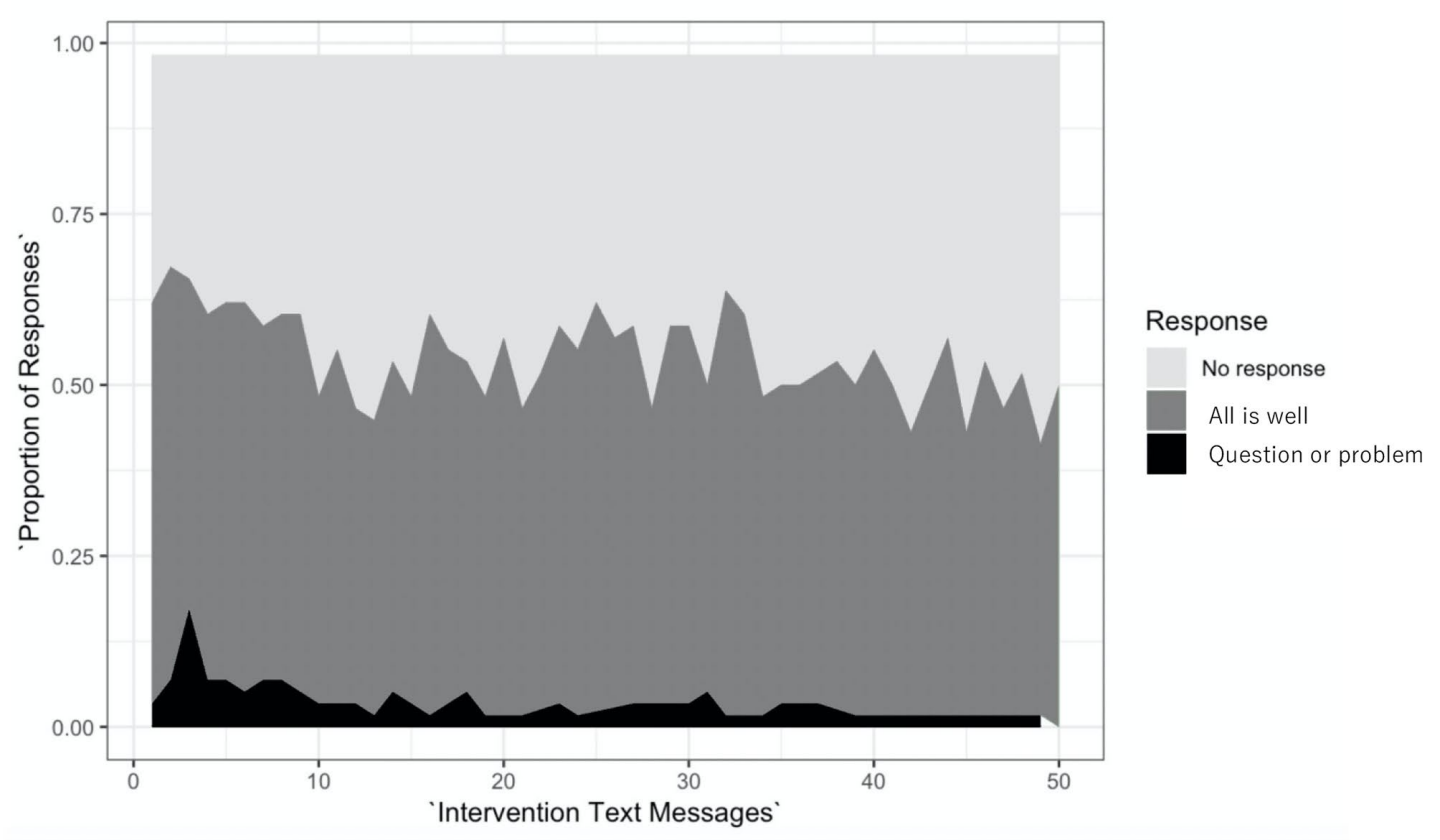
Proportion of responses to study text messages over the six month intervention period

Participant-level feasibility was assessed by the rate of response for text messages. Each woman in the intervention arm was sent 50 text messages during the six-month study period. All intervention participants responded to at least one message. The average overall response rate was 1,595/2,900 (55%). The response rate decreased slightly over time, with 402/638 (63%) messages eliciting a response during the first month compared to 191/406 (47%) messages eliciting a response during month six (Fig. 2). Thirty-nine of 66 women (59%) in the intervention arm asked at least one question during the intervention.

Interviews focused on participant-level acceptability were conducted with 91 of 106 women who completed the study, including 48/50 (96%) intervention and 43/56 (77%) control participants. Overall, women reported feeling highly motivated to adhere to ART for their health (36%, 33/91), children (22%, 20/91), and longevity (20%, 18/91). All women reported that the clinic cared about their wellbeing, with several women mentioning that they felt like they “received good attention,” and were appreciative of staff “provid[ing] hope” and “encouragement.”

Among intervention participants, feedback about the intervention content was overwhelmingly positive. One woman summarized: “The messages [I] was receiving gave me hope. I felt I was not alone.” All intervention participants felt that the text messages improved their overall wellbeing and ART adherence, indicating that they “helped motivate me to take my drugs” and “reduced [the] stress [of taking ART].” When asked what message content was most meaningful, the most common responses included religion (10/48, 20%), children and families (6/48, 13%), and support from clinic staff (3/48, 6%). For example, “The messages telling me I am important and needed in the lives of my children and being told I am beautiful motivate me a lot.” None of the women expressed dissatisfaction about the message content and all stated that they found the intervention helpful. Five (10%) women had suggestions for making the content of the text messages more meaningful and increasing the effectiveness of the intervention. Two (4%) suggested increasing the religious messaging and two (4%) suggested that “messages should be sent only on weekdays” because “if you asked a question on Saturday…you have to wait until Monday [to get a call].”

The structure of the intervention was generally well-received. Forty-four (92%) participants indicated that the message frequency was “good,” while two (4%) suggested there were too few messages and two (4%) suggested there were too many. Six (12%) women reported difficulty responding to messages due to lack of airtime credit. Three (6%) women felt that nurses took too long to call them back when they had a question. One said, “When I ask or reply with a question, I would prefer to talk then and there so that if there is a problem it is solved immediately.” In addition, two (4%) women suggested that nurses should respond to questions via text.

Women consistently reported that they appreciated the measures taken to protect their confidentiality. For example, one woman reported that referring to ART as blood pressure medication “guaranteed confidentiality.” Sixteen (33%) women in the intervention arm volunteered that at least one person was curious about their study text messages, but there was no inadvertent disclosure. To ensure confidentiality, one woman shared that she deleted the text messages after reading them.

## Discussion

This RCT of a theory-based, mHealth intervention aimed to support viral suppression in women initiating or changing ART regimens. Despite efforts to extend the recruitment period and expand inclusion criteria to include women who did not report engagement in sex work, only 119 of the intended 210 women were enrolled, limiting our ability to detect significant findings. Recruitment was challenging because it was difficult to find a sufficient number of women who were HIV-seropositive, not currently taking ART, and willing to initiate treatment. In a setting where public-sector HIV treatment had been available since 2004 and treatment for all people living with HIV regardless of CD4 count or disease stage was implemented in 2016, this meant that most recruitment had to be achieved by identifying and enrolling FSW who were newly diagnosed with HIV into the Mombasa Cohort. We also recruited women who were switching to a second-line regimen due to treatment failure, but with modern ART regimens, these cases were infrequent. Of the 119 women who enrolled in the study, 69% of intervention and 63% of control participants were virally suppressed at six months. Participants in the intervention arm reported significantly higher adherence compared to participants in the control arm during the first month of the intervention.

The small magnitude of the overall difference in viral suppression between the study arms may be related to the inclusion of an active control condition. Specifically, the standard of care included monthly appointments to monitor and support ART adherence and problem solve adherence barriers. Since both are evidence-based strategies for ART adherence [27], it is possible the active control attenuated the observed effect of *Motivation Matters!*.

A secondary analysis was conducted to identify the intervention effect within the intended participant population. In addition to relaxing the inclusion criteria to include women who did not engage in sex work, 20% of the enrolled women denied taking ART, but had an undetectable viral load at baseline. Among women who reported engaging in sex work and who had a detectable viral load at baseline, viral load suppression was achieved by 74% of intervention participants compared to 46% of control participants.

While mHealth interventions have demonstrated some efficacy for changing behavior, there are gaps in understanding the mechanisms through which these interventions effect change [7]. In the present trial, intervention participants reported modestly, though non-significantly, higher levels of information, motivation and behavioral skills for ART adherence compared to participants in the control arm, which is consistent with other studies [28].

Participants receiving *Motivation Matters!* responded to 55% of the intervention messages, suggesting high individual-level feasibility. This is lower than the 70% response rate in the first published 2-way mHealth ART adherence intervention in 2010 [8], but much higher than the 28% average response rate in contemporary two-way mHealth interventions for PWH in Africa [29]. It is possible that text messages simply are not as novel as they were when the original trials were conducted. Spam text messages have become ubiquitous and phone users may be accustomed to ignoring messages. It may be necessary to adapt mHealth interventions to align with people’s changing interactions with technology to maintain their novelty and relevance.

In the decade since the initial 2-way text message intervention showed improvements in viral suppression [8], subsequent trials have demonstrated inconsistent effects [29, 30]. The timing of mHealth interventions in relation to individuals’ HIV treatment history may be an important contextual factor. Interventions that coincide with ART initiation or regimen changes following virologic failure could be more impactful than interventions implemented after the habit of taking a new ART regimen has been established [31].

The qualitative data collected at the end of the study provided useful information about participant-level acceptability of the intervention and identified points for improvement. The content, number, and frequency of intervention text messages were generally well-received. The favorable feedback on acceptability may have extended from development of the intervention based on focus group discussions with the target population [21].

This study had several notable strengths. First, it was the first intervention designed by and for FSW, a key population in the HIV epidemic. Second, validated tools were used to explore potential mechanisms influencing ART adherence and viral suppression. The LW-IMB-AAQ allowed a preliminary examination of the intervention’s effects on information, motivation, and behavioral skills [12, 16]. Third, the focus on viral suppression, a biological outcome, added methodological rigor and a critically important clinical endpoint. Finally, the data demonstrating the feasibility and acceptability of this intervention for the target population provide a strong foundation for a larger study.

This study also had important limitations. First, the trial did not reach its target sample size, and the resulting analyses were under-powered to detect an intervention effect. Second, while the intervention was developed for FSW, inclusion criteria were relaxed to increase the number of evaluable participants. The intervention effect was larger in a subset analysis including only FSW, suggesting the potential for greater impact in the population for which the intervention was designed. Third, when viral load testing was performed after completion of the trial, 20% of participants were unexpectedly virally suppressed at enrollment. This suggests that some women who reported that they were just starting ART were already on treatment. Finally, while participants received sufficient paid airtime to return messages, several women reported that they ran out of airtime to respond. Future studies may benefit from providing free texting to the study number and utilization of community-owned wireless networks in Africa that provide free services to members.

## Conclusion

Overall, *Motivation Matters!* achieved 6% higher viral suppression at six months compared to the control arm, though the difference was not statistically significant. The effect size was much larger in FSW who were not virally suppressed at baseline, the population for which the intervention was developed. A larger multi-site trial focused exclusively on this population is needed to demonstrate effectiveness at scale and examine barriers and facilitators to widespread implementation.

## Data Availability

This study was conducted with approval from the Kenyatta National Hospital - University of Nairobi Ethics and Research Committee (KNH-UON ERC), which requires that we release data from Kenyan studies (including de-identified data) only after they have provided their written approval for additional analyses. As such, data for this study will be available from the authors upon request, with written approval for the proposed analysis from the KNH/UON ERC. Their application forms and guidelines can be accessed at http://erc.uonbi.ac.ke/. To request these data, please contact KRTC Administrator at ude.wu@seraynek.

## References

[1] UNAIDS. Understanding Fast-Track: accelerating action to end the AIDS epidemic by 2030. 2015. www.unaids.org/sites/default/files/media_asset/201506_JC2743_Understanding_FastTrack_en.pdf.

[2] Ramjee G, Daniels B. Women and HIV in Sub-Saharan Africa. AIDS Res Ther. 2013 Dec 13;10(1):30. 10.1186/1742-6405-10-30. PMID: 24330537; PMCID: PMC3874682 10.1186/1742-6405-10-30 PMC3874682 24330537

[3] Baral S, Beyrer C, Muessig K, Poteat T, Wirtz AL, Decker MR, Sherman SG, Kerrigan D. Burden of HIV among female sex workers in low-income and middle-income countries: a systematic review and meta-analysis. Lancet Infect Dis. 2012 Jul;12(7):538–49. 10.1016/S1473-3099(12)70066-X. 10.1016/S1473-3099(12)70066-X 22424777

[4] Horvath T, Azman H, Kennedy GE, Rutherford GW (2012). Mobile phone text messaging for promoting adherence to antiretroviral therapy in patients with HIV infection. Cochrane Database of Syst Rev.

[5] Comulada WS, Wynn A, van Rooyen H, Barnabas RV, Eashwari R, van Heerden A (2019). Using mHealth to deliver a home-based testing and counseling program to improve linkage to care and ART adherence in rural South Africa. Prev Sci.

[6] Gouse H, Robbins RN, Mellins CA (2018). Empowering lay-counsellors with technology: Masivukeni, a standardized multimedia counselling support tool to deliver ART counselling. AIDS Behav.

[7] Simoni JM, Ronen K, Aunon FM (2018). Health behavior theory to enhance eHealth intervention research in HIV: Rationale and review. Curr HIV/AIDS Rep.

[8] Lester RT, Ritvo P, Mills EJ et al. Effects of a mobile phone short message service on antiretroviral treatment adherence in Kenya (WelTel Kenya1): a randomised trial. Lancet. 2010 Nov27;376(9755):1838–45. 10.1016/S0140-6736(10)61997-6 21071074

[9] Pop-Eleches C, Thirumurthy H, Habyarimana JP (2011). Mobile phone technologies improve adherence to antiretroviral treatment in a resource-limited setting: a randomized controlled trial of text message reminders. AIDS.

[10] Thakkar J, Kurup R, Laba TL (2016). Mobile telephone text messaging for medication adherence in chronic disease: a meta-analysis. JAMA Intern Med.

[11] Lancaster KE, Cernigliaro D, Zulliger R, Fleming PF (2016). HIV care and treatment experiences among female sex workers living with HIV in sub-saharan Africa: a systematic review. Afr J AIDS Res.

[12] Fisher JD, Fisher WA, DiClemente R, Crosby R, Kegler R (2002). The information-motivation-behavioral skills model. Emerging Promotion Research and Practice.

[13] McClelland RS, Richardson BA, Cherutich P (2015). A 15-year study of the impact of community antiretroviral therapy coverage on HIV incidence in kenyan female sex workers. AIDS.

[14] McClelland RS, Graham SM, Richardson BA, Peshu N, Masese LN, Wanje GH, Mandaliya KN, Kurth AE, Jaoko W, Ndinya-Achola JO. Treatment with antiretroviral therapy is not associated with increased sexual risk behavior in Kenyan female sex workers. AIDS. 2010 Mar 27;24(6):891–7. doi: 10.1097/QAD.0b013e32833616c7. PMID: 20179576; PMCID: PMC2853894. 10.1097/QAD.0b013e32833616c7 PMC2853894 20179576

[15] Lu M, Safren SA, Skolnik PR (2008). Optimal recall period and response task for self-reported HIV medication adherence. AIDS Behav.

[16] Oyugi JH, Byakika-Tusiime J, Charlebois ED (2004). Multiple validated measures of adherence indicate high levels of adherence to generic HIV antiretroviral therapy in a resource-limited setting. J Acquir Immune Defic Syndr.

[17] The LifeWindows Project Team. The LifeWindows information motivation behavioral skills ART Adherence Questionnaire (LW-IMB-AAQ). Center for Health, Intervention, and Prevention. University of Connecticut; 2006.

[18] Dubov A, Altice FL, Fraenkel L (2018). An information–motivation–behavioral skills model of PrEP uptake. AIDS Behav.

[19] Graham SM, Micheni M, Secor A (2018). HIV care engagement and ART adherence among kenyan gay, bisexual, and other men who have sex with men: a multi-level model informed by qualitative research. AIDS Care.

[20] Torija CST, Vázquez GV, Montijo SSR, Romo LDLE (2015). The information and motivation and behavioral skills model of ART adherence among HIV-positive adults in Mexico. J Int Assoc Provid AIDS Care.

[21] Aunon FM, Okada E, Wanje G (2020). Iterative development of an mHealth intervention to support antiretroviral therapy initiation and adherence among female sex workers in Mombasa, Kenya. J Assoc Nurses AIDS Care.

[22] Odeny TA, Hughes JP, Bukusi EA (2019). Text messaging for maternal and infant retention in prevention of mother-to-child HIV transmission services: a pragmatic stepped-wedge cluster-randomized trial in Kenya. PLoS Med.

[23] Odeny TA, Bailey RC, Bukusi EA (2012). Text messaging to improve attendance at post-operative clinic visits after adult male circumcision for HIV prevention: a randomized controlled trial. PLoS ONE.

[24] Iyer P, Mwai D. N’ganga A. costing Kenya’s current and proposed HIV testing and counseling algorithms. Futures Group National AIDS and STI Control Programme; February; 2013.

[25] Graham SM, Masese L, Gitau R (2010). Antiretroviral adherence and development of drug resistance are the strongest predictors of genital HIV-1 shedding among women initiating treatment. J Infect Dis.

[26] Huh D, Flaherty BP, Simoni JM (2012). Optimizing the analysis of adherence interventions using logistic generalized estimating equations. AIDS Behav.

[27] Consolidated Guidelines on the Use of Antiretroviral Drugs for. Treating and preventing HIV infection: recommendations for a Public Health Approach. Geneva: World Health Organization; 2013 Jun. p. 24716260. 24716260

[28] Chang SU, Choi S, Kim S, Song M (2014). Intervention strategies based on information-motivation-behavioral skills model for health behavior change: a systematic review. Asian Nurs Res.

[29] Linnemayr S, Huang H, Luoto J, Kambugu A, Thirumurthy H, Haberer JE (2017). Text messaging for improving antiretroviral therapy adherence: no effects after 1 year in a randomized controlled trial among adolescents and young adults. Am J Public Health.

[30] Shet A, De Costa A, Kumarasamy N, Rodrigues R, Rewari BB, Ashorn P (2014). Effect of mobile telephone reminders on treatment outcome in HIV: evidence from a randomised controlled trial in India. BMJ.

[31] Sikkema KJ, Abler L, Hansen NB, Wilson PA, Drabkin AS, Kochman A (2014). Positive choices: outcomes of a brief risk reduction intervention for newly HIV-diagnosed men who have sex with men. AIDS Behav.

